# Letter the editor: serious methodological concerns about a recently published meta-analysis on oxygen therapy

**DOI:** 10.1186/s40560-021-00573-5

**Published:** 2021-12-07

**Authors:** Thomas Lass Klitgaard, Olav Lilleholt Schjørring, Frederik Mølgaard Nielsen, Christian Sylvest Meyhoff, Marija Barbateskovic, Jørn Wetterslev, Anders Perner, Bodil Steen Rasmussen

**Affiliations:** 1 Department of Anaesthesia and Intensive Care, Aalborg University Hospital, Aalborg, Denmark; 2 Department of Clinical Medicine, Aalborg University, Aalborg, Denmark; 3 Department of Anaesthesia and Intensive Care, Bispebjerg and Frederiksberg Hospital, University of Copenhagen, Copenhagen, Denmark; 4 Department of Intensive Care, Rigshospitalet, Copenhagen University Hospital, Copenhagen, Denmark; 5 Copenhagen Trial Unit, Centre for Clinical Intervention Research, Rigshospitalet, Copenhagen University Hospital, Copenhagen, Denmark; 6 Collaboration for Research in Intensive Care, Rigshospitalet, Copenhagen University Hospital, Copenhagen, Denmark

**Keywords:** Oxygen, Critical care, Systematic review, Meta-analysis

## Abstract

In a recent paper, Chen et al. report the findings of a systematic review with meta-analysis concerning conservative versus conventional oxygen therapy for critically ill patients. We wish to commend the authors for their interest in the matter. However, the authors appear to misquote findings, fail to report results for all specified analyses, do not identify all relevant trials, have post hoc changed the eligibility criteria, and have seemingly switched directions of effects in analyses of secondary outcomes. These issues have led to incorrect conclusions concerning the effects of targeted oxygen therapy in critically ill patients.

To the editor,

We have with interest read the systematic review with meta-analysis concerning the effects of conservative versus conventional oxygen therapy for critically ill patients by Chen et al. [1]. However, we have several concerns relating to the methodology and findings. None of the analyses and figures presented in this letter have been published elsewhere. They were specifically constructed for the purpose of this letter.

In the paper by Chen et al. [1], the mortality rates are erroneously quoted from several trials in the meta-analysis of mortality at longest follow-up. In the paper by Schjørring et al. [2], a mortality of 514/1447 and 529/1441 in the higher and lower group is incorrectly quoted. The correct mortality was 613/1447 and 618/1441, respectively [2]. Mortality in the liberal group in the study by Barrot et al. was 31/102 [3], not 39/102 as stated. Twenty-eight-day mortality for Asfar et al. is quoted despite 90-day mortality is reported in the trial paper [4]. The ICU-mortality in the modified intention-to-treat population for Girardis et al. is quoted although hospital mortality for the intention-to-treat cohort is reported in the trial paper [5]. A revised meta-analysis is presented in Fig. 1. Chen et al. reported the RR as 1.01 (95% CI 0.94–1.09), so there is a slight difference in the 95% CI [1].

**Fig. 1:**
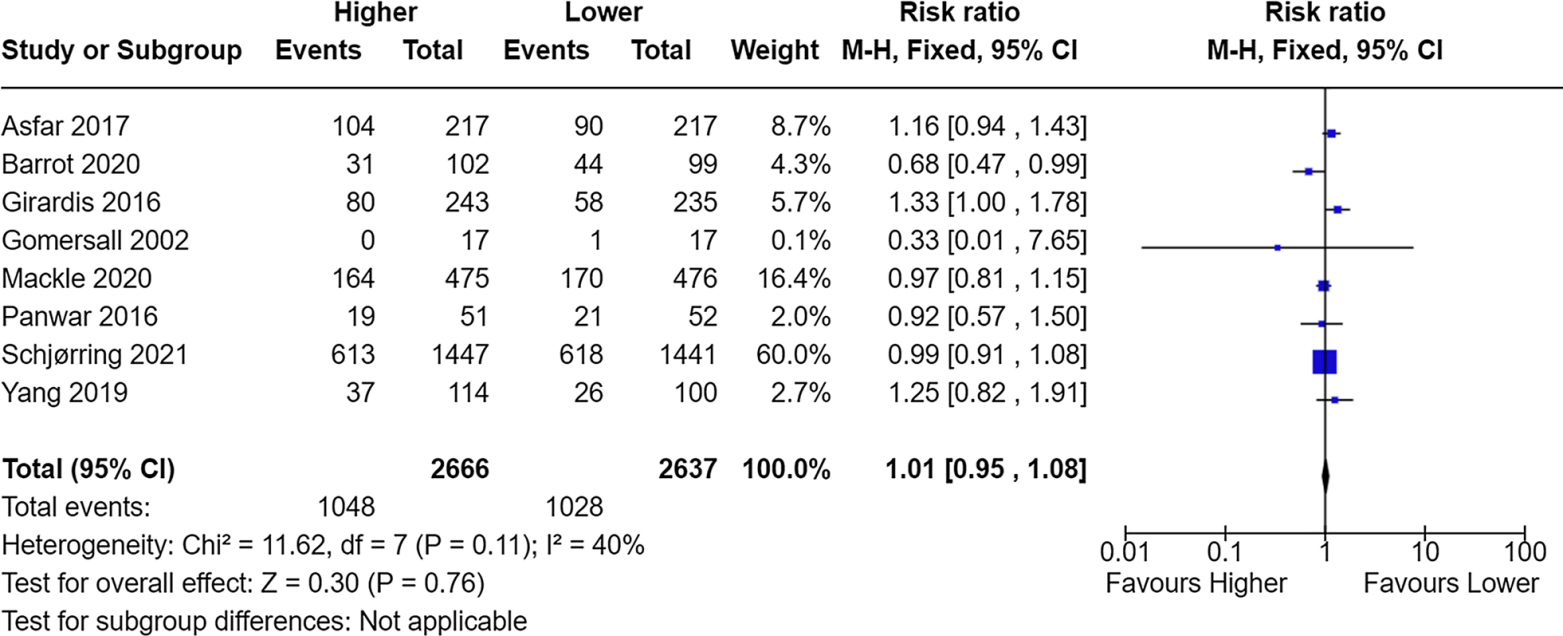
Meta-analysis of mortality at the longest follow-up. M-H, Fixed denotes Mantel–Haenszel (M-H) fixed-effect model, CI confidence interval

The literature search is insufficient as the authors fail to identify four relevant papers focusing on: ICU-patients with acute exacerbation of chronic obstructive pulmonary disease [6]; oxygen therapy after cardiac-arrest [7]; normobaric oxygen in stroke patients [8]; and hyperoxaemia in stroke patients [9]. The first paper should have been identified and included in the meta-analysis, whilst the latter three should have been identified and excluded as per their stated exclusion criteria [1]. In their PRISMA-diagram, the authors state that six trials were excluded after full-text review and present the trials along with reasons for exclusions (Additional file 3: Table S1). In the main text and in this table only five trials are quoted. Moreover, the eligibility criteria have been changed post hoc, without justification, now excluding trials with patients at risk of ischaemia or hypoxic encephalopathy. No such criteria are mentioned in the protocol [10].

The authors’ choice of subgroup analysis based on baseline ratios of partial pressure of oxygen to fraction of inspired oxygen (PaO_2_/FiO_2_) as according to mild, moderate, and severe acute respiratory distress syndrome (> 200 mmHg, 100–200 mmHg, and < 100 mmHg, respectively) is problematic, as the results from this analysis, specified in the statistical analysis section, are not presented, except for the results from the sensitivity analysis of trials excluding patients with a PaO_2_/FiO_2_ ratio < 100 mmHg (in the abstract). In the main text and their Fig. 2, the authors pool three trials all excluding patients with baseline PaO_2_/FiO_2_ ratios < 100 mmHg [4, 11] or < 150 mmHg [5]. This selection is inappropriate, as the approximate mean ratios in Mackle et al. were 252 mmHg [12], and in Panwar et al. 247 mmHg [13]. Though both trials did not restrict inclusion based on PaO_2_/FiO_2_ ratios, most patients included in these two trials clearly satisfy the criteria for inclusion in the subgroup analysis above. As no baseline PaO_2_/FiO_2_ ratios were presented by Girardis et al. [5], no knowledge of severity of respiratory failure can be ascertained. Therefore, this study should be excluded from the subgroup analysis. In the HOT-ICU trial [2], inclusion was not restricted by PaO_2_/FiO_2_ ratio, and the median baseline PaO_2_/FiO_2_ ratios were approximately 118 mmHg in both groups. However, a substantial proportion of patients had a ratio ≥ 150 mmHg. We acknowledge that cohort-level-based separations may seem to provide easy new knowledge when performing a meta-analysis, but with such heterogenous groups of included patients in each trial, the only reliable answer to risks according to baseline degree of respiratory failure would come from individual-based-separations and access to all trials’ datasets. Below is provided a revised meta-analysis on mortality at longest follow-up stratified on the specified separation of trials (Fig. 2). This clearly changes the conclusion of the subgroup analysis, as the subgroup of trials with reported baseline PaO_2_/FiO_2_ ratios > 200 mmHg now produces a statistically non-significant result (and non-significant test for subgroup differences), contrary to the results presented by the authors.

**Fig. 2:**
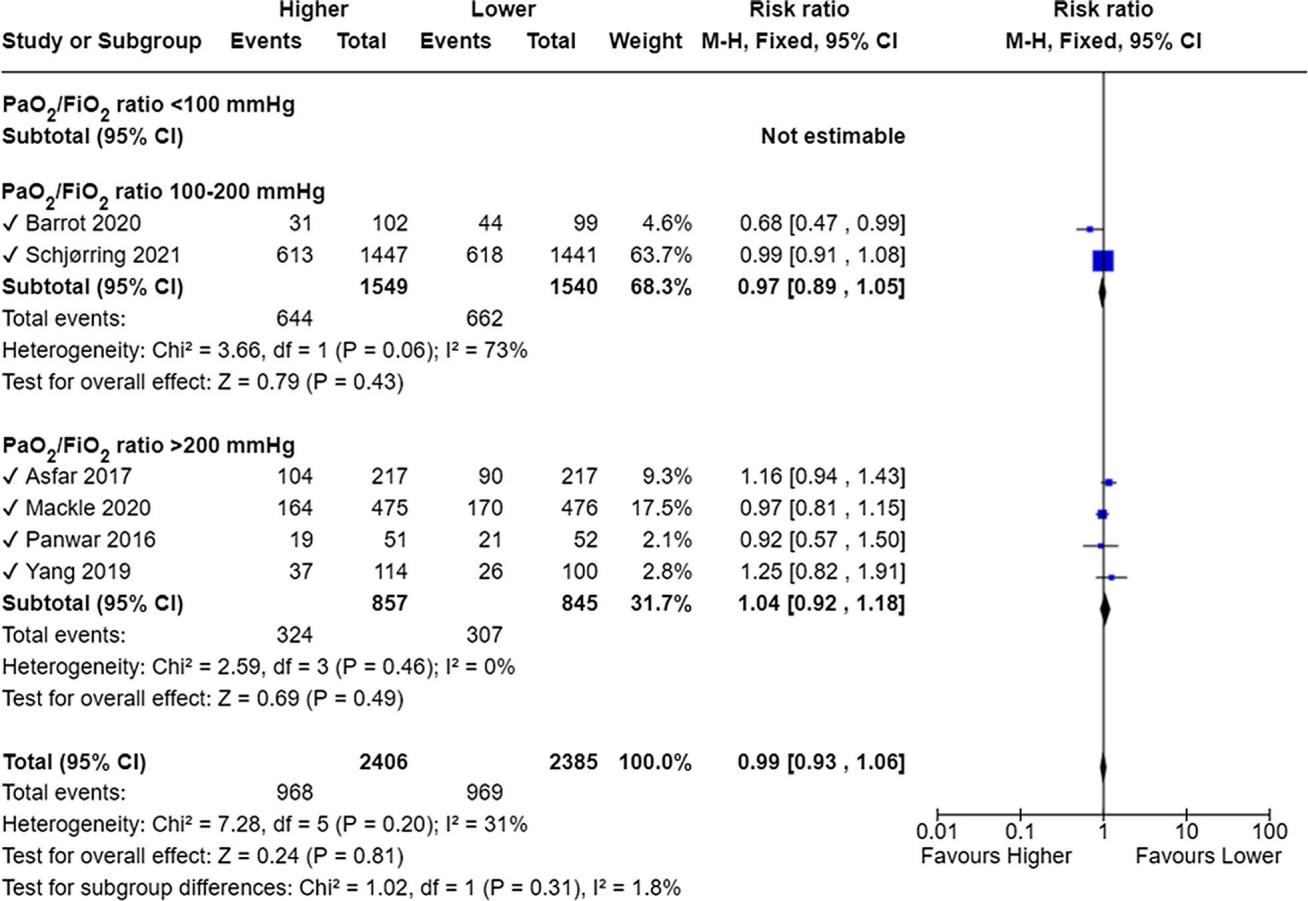
Meta-analysis of mortality at the longest follow-up, separating trials as according to reported baseline PaO_2_/FiO_2_ ratios. M-H, Fixed denotes Mantel–Haenszel fixed-effect model, CI confidence interval

Lastly, it appears that the two compared groups have been switched when reporting serious adverse events, despite correct findings are provided in the supplement (Additional file 6) [1]. If inversed, the results are in line with the meta-analysis provided below (Figs. 3, 4, 5). Conclusions based on these analyses now point in the opposite direction as to what was reported by authors, though still statistically insignificant.

**Fig. 3:**
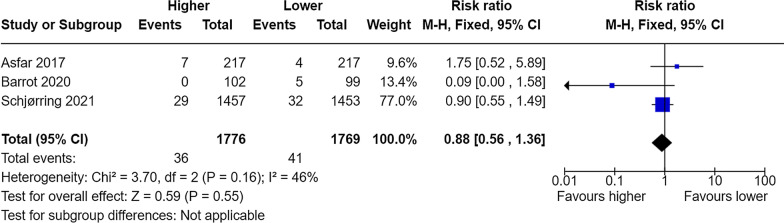
Meta-analysis of mesenteric ischaemia at longest follow-up. M-H, Fixed denotes Mantel–Haenszel fixed-effect model, CI confidence interval. Chen et al. reported the RR for mesenteric ischaemia as 1.15 (95% CI 0.73–1.19)

**Fig. 4:**
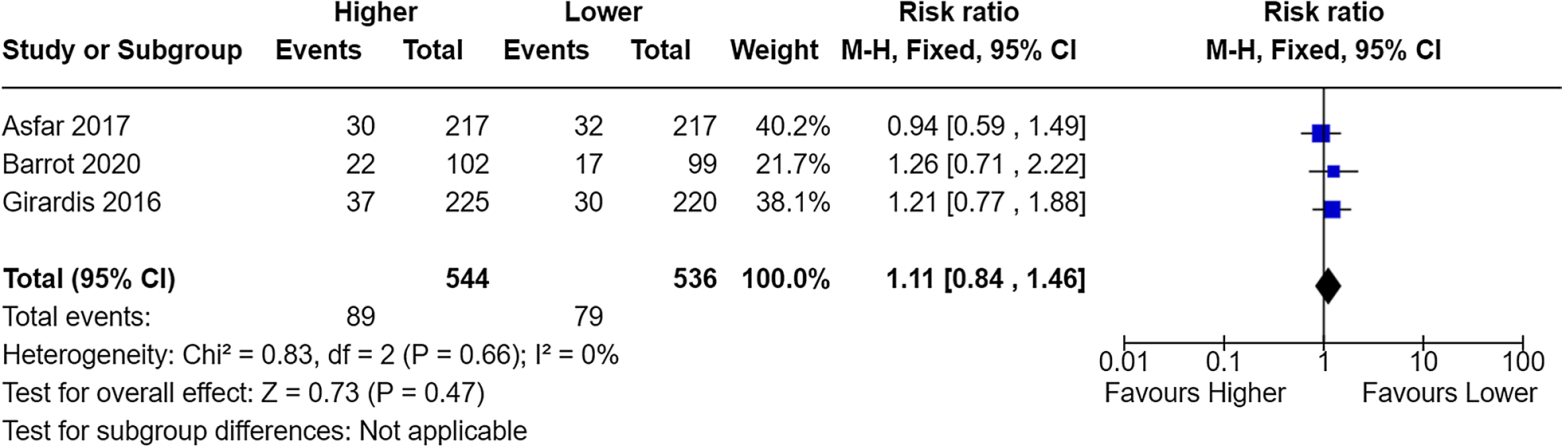
Meta-analysis of pneumonia at longest follow-up. M-H, Fixed denotes Mantel–Haenszel fixed-effect model, CI confidence interval. Chen et al. reported the RR for pneumonia as 0.92 (95% CI 0.72–1.18)

**Fig. 5:**
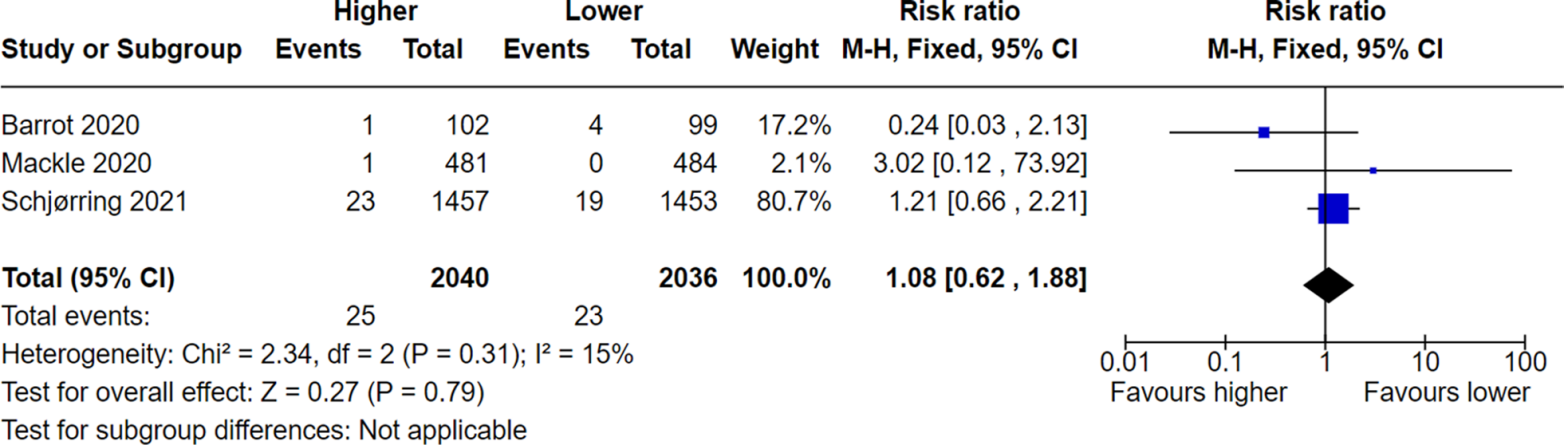
Meta-analysis of stroke at longest follow-up. M-H, Fixed denotes Mantel–Haenszel fixed-effect model, CI confidence interval. Chen et al. reported the RR for stroke as 0.93 (95% CI 0.53–1.63)

Meta-analyses of high-quality trials are considered the highest level of evidence. Thus, the methodology applied needs to be of similar high quality. If not, inappropriate conclusions may be drawn, potentially misguiding clinical practice. In their review and meta-analysis, Chen et al. fail in several crucial domains, thereby presenting incorrect results and conclusions.

## Data Availability

Not applicable.

## Abbreviations

CI: Confidence interval
FiO_2_: Fraction of inspired oxygen
HOT-ICU trial: Handling Oxygenation Targets in the Intensive Care Unit trial
M-H, Fixed: Mantel–Haenszel fixed-effect model
mmHg: Millimetres of mercury
PaO_2_: Partial pressure of arterial oxygen
